# Impact of Chemicals on the Age of Menarche: A Literature Review

**DOI:** 10.3390/children10071234

**Published:** 2023-07-17

**Authors:** Xristos Anastasiadis, Alkis Matsas, Theodoros Panoskaltsis, Panagiotis Bakas, Dimitrios T. Papadimitriou, Panagiotis Christopoulos

**Affiliations:** Second Department of Obstetrics and Gynecology, “Aretaieion” Hospital, Faculty of Medicine, National and Kapodistrian University of Athens, 11528 Athens, Greece

**Keywords:** menarche, chemicals, endocrine disruptors, environmental pollution, air pollution

## Abstract

A growing body of evidence suggests that chemicals interfere with the age of onset of menarche. We conducted a review in order to demonstrate the relationship between several categories of chemicals and menarche. We searched for English language papers using the Medline/PubMed database until April 2023. The chemical factors found to affect menarche were prenatal and antenatal smoke, phthalates, phenols, organochlorines, perfluoroalkyls and polyfluoroalkyls, metals, air pollutants and polybrominated diphenyl ethers. Low or high exposure to each chemical compound could affect the age of menarche, leading to early or delayed menarche. Furthermore, the results show that intrauterine exposure may have a different impact from antenatal exposure. There is evidence that endocrine-disrupting chemicals affect the age of menarche, but more research needs to be conducted.

## 1. Introduction

Menarche represents an indicator of women’s health, the degree of maturation and fertility. Menarcheal age seems to be affected by race, socioeconomic status and genetic background. The USA population experiences menarche at a median age of 11.9 years, whereas the French population experiences it at 12.8 years old [1,2]. In Canada, adolescents have menarche at 12.67 years, whereas in China, girls have it at the age of 12.3 years [3,4]. With the passage of time, changes in the age of onset of menarche have been observed. Bio-based materials from 2.5 million to a thousand years ago indicate that the age of menarche ranged between 7 and 13 years. In the 19th century and the first half of the 20th century, after the deterioration of the quality of life and hygiene, diseases were easily transmitted and as a result there was a retardation in the development of the children, thus an increase in the age of menarche occurred; specifically, it was noted as 15–16 years of age. Subsequently, along with the improvements in socioeconomic conditions in the second half of the 20th century, a decrease in the age of first menstruation was noted [5]. Over the years, the increasing need for a more convenient lifestyle has exposed humans to a wide range of chemicals, such as phthalates, phenols, metals, smoke substances and organochlorines. These chemicals, which are known to mimic the functions of hormones and have been demonstrated to induce the development of diseases, are called endocrine-disrupting compounds (EDCs). EDCs are either natural or synthetic exogenous materials that interfere with the physiology of reproduction and growth. The first reference of the effect of chemicals on the reproductive health of women was in 1971, when the use of diethylstilbestrol (DES) to prevent miscarriages was forbidden because of a publication that showed an increase in rare cervicovaginal cancers in women treated with DES [6]. The concerns about EDCs grew with their extensive use in building materials, vehicles, furniture, plastics, and products such as baby feeding bottles, the lining in tin food containers and children’s toys [7]. This review aims to investigate the effects of EDCs on the occurrence of menarche.

## 2. Materials and Methods

**Figure 1:**
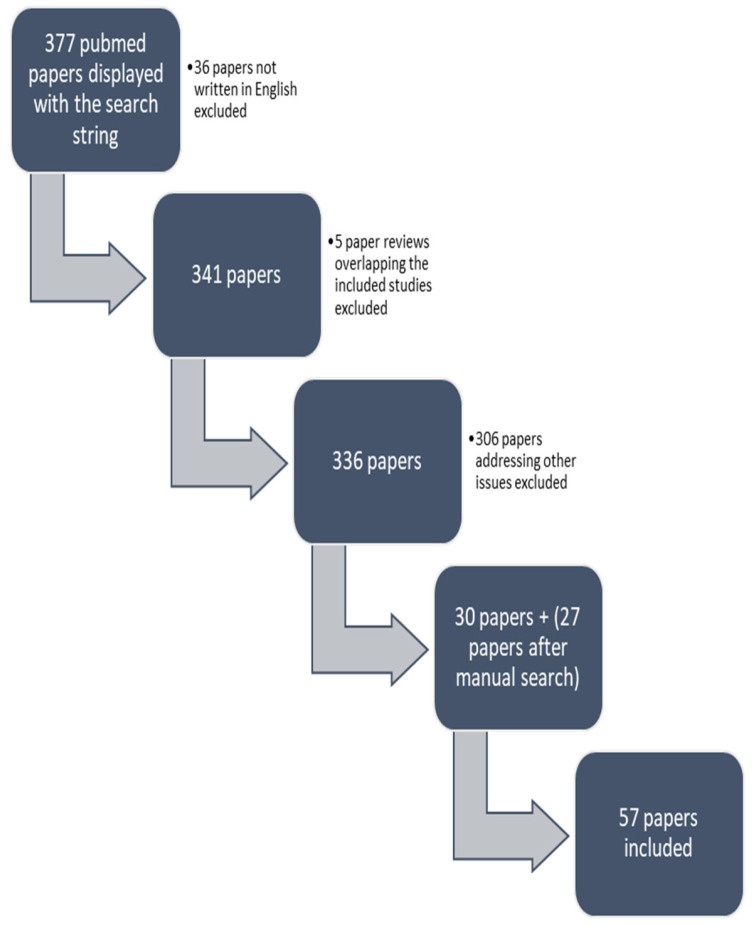
Selection of papers.

In order to identify the chemical factors acting during the intrauterine period and childhood that are potentially related to the onset of menarche, the authors conducted an extensive search in Medline/PubMed in the English language up until April 2023. The following search string was used: chemicals OR “environmental pollution” OR “endocrine disruptors” OR “air pollution” AND menarche. In total, 377 results were displayed. A total of 36 papers not written in English were excluded, 5 reviews overlapping with the included studies were excluded, and 306 papers addressing other issues were also excluded. From the search string, 30 papers were included. After an additional manual search and an extension of the search to the reference lists of all retrieved articles, 57 papers were finally included in our review (Figure 1). The types of studies included were prospective, retrospective, cross-sectional and case–control studies and one review [8,9,10,11,12,13,14,15,16,17,18,19,20,21,22,23,24,25,26,27,28,29,30,31,32,33,34,35,36,37,38,39,40,41,42,43,44,45,46,47,48,49,50,51,52,53,54,55,56,57,58,59,60,61,62,63,64] (Table S1).

## 3. Results

### 3.1. Smoke Exposure

There are indications that there are effects from tobacco on the female reproductive system. Lutterodt et al. demonstrated a change in the number of ovarian cells after smoke exposure. The authors studied 28 first-trimester female fetuses from legal abortions. The tissues of the ovaries of the fetuses were dissected and analyzed using a stereomicroscope. The predominant metabolite of nicotine, cotinine, was measured in maternal urine samples to assess exposure to smoke. It was found that the ovaries of the embryos that were exposed to maternal cigarette smoke had reduced somatic cells, and there was no difference in the number of oogonia compared to the fetal ovaries that were not exposed. The interaction between somatic cells and oogonia is necessary for the development of the ovaries and oocytes; therefore, prenatal nicotine exposure likely influences the ovarian reserve of females [65]. In a meta-analysis by Yermachenko et al., which included 17 studies from 1995 to 2014, the association between prenatal exposure to smoke and the age of menarche was examined. In 13 studies (10 prospective cohorts and 3 cross-sectional), the age of menarche was a continuous variable, while in 5 studies (4 cohorts and 1 cross-sectional), it was a qualitative variable (ages of 11 or below, 12 or below, 13 or above and 15 or above). The meta-analysis showed a positive relationship between tobacco exposure and the age of menarche (−0.092 years 95% CI: −0.160, −0.024 years). In the studies where menarche was recorded as a categorical variable, girls exposed in utero to smoke had a 15% increased likelihood of experiencing menarche before 11 years of age [12]. A retrospective study of 751 girls in Shanghai reported a 1.84 times increased odds of early menarche after intrauterine exposure to maternal passive smoking after adjustment for maternal menarche and childbirth weight. The authors also reported a shorter cycle length in the exposed group [8]. A study of 1493 girls in Australia investigated the effect of prenatal exposure to smoke on menarche. The study included mothers who smoked on most days of the gestational period, those who smoked on some days and those who had never smoked cigarettes. Daughters exposed to cigarettes on most days of the pregnancy had a hazard ratio of 1.4 for early menarche [9]. In 2018, Houghton et al. investigated why studies disagree regarding the effect of prenatal smoke on daughters’ menarche. They investigated how the height and weight of daughters to mothers who smoked during pregnancy, in the 4th month, 1st year and 4th year of life, influenced menarche time. The growth of children can be rapid, stable or slow according to the Centers of Disease Control (CDC) patterns. Rapid refers to when a child increases by two major reference percentiles of weight from the day of birth to the 4th year of life, slow refers to when a child drops by two major percentiles and stable refers to when a child remains within two major percentiles in this period. In particular, the authors found that rapid growth decreased the onset of menarche by 0.56 years and that slower growth resulted in menarche occurring 0.53 years later [10]. Windham et al. assessed both prenatal and antenatal smoke exposure. Intrauterine tobacco exposure was divided into four categories, namely, no smoking, 1–9 cigarettes, 10–19 cigarettes and 20 plus cigarettes, and childhood exposure was divided into three groups, namely, no smoking, 0–8 pack-years and 8 plus pack-years, assuming both maternal and paternal smoking. A number of 20 cigarettes or more in the prenatal period reduced the age of menarche of adolescents by 0.22 years (CI −0.49, 0.05), and in the non-white population, menarche occurred approximately 6 months earlier. In addition, girls whose mothers were heavy smokers during pregnancy (>20 cigarettes) and whose parents had 8 pack-years or more in the childhood period had a mean age of menarche of 12.75 years, while non-exposed girls had a mean age of menarche of 13.08 years. Girls exposed to 8 pack-years or more of environmental tobacco smoke had menarche 0.15 years earlier, while both in utero exposure and childhood exposure were related to menarche occurring 4 months earlier [11]. Work by Kang et al. showed the effects of antenatal smoke, including passive and active smoking, on menarche. Girls with secondhand smoke exposure were assigned to a ‘never exposed’, ‘light exposure’ or ‘heavy exposure’ group. The results were adjusted for the region of residence, family income, physical activity, alcohol consumption and BMI. Those who were passively exposed to the smoke of their parents had a 1.12 times (95% CI: 1.05, 1.19) increased likelihood of reaching menarche than those who were not exposed. Interestingly, girls who smoked before the 12th year of their life had menarche 1.68 times earlier than those who never smoked [13]. A study of 20,061 females in southern China investigated the effect of the number of smokers in the family environment, as well as the frequency of smoke exposure, on the age of menarche. The timing of exposure was divided into <5 days per week and ≥5 days/week, and the number of people who smoked in the house was none, one, two or more. In particular, secondhand smoke from two or more people in the house resulted in menarche occurring 0.38 years earlier, and an exposure of 5 days or more in a week decreased the age of menarche by 0.19 years [14]. Another study examined both prenatal and antenatal exposure. Information regarding prenatal smoke exposure was provided by the exposure of the pregnant women throughout the gestational period which was categorized into four levels: no exposure, 1–9 (light), 10–19 (medium) and 20+ (heavy). After adjustment for birthweight and postnatal growth patterns, Ferris et al. demonstrated that secondhand smoke increased the likelihood of not reaching menarche by 2.1 times. Furthermore, when girls were exposed to smoke both prenatally and antenatally, a delay in the age of menarche was observed (OR: 2.2, 95% CI: 1.1, 4.6) [15]. In summary, the majority of the above studies demonstrate an earlier menarche after exposure to smoke either prenatally or antenatally.

### 3.2. Phthalates

Phthalates are stabilizers and plasticizers in commonly used household products such as personal care products, food packaging, medical equipment, toys and building materials [66]. In cosmetics and personal care products, phthalates are used for their oily texture and ability to impart flexibility to thin films (e.g., in mascara and nail polish) [67]. These chemicals can be absorbed through the skin (cream solutions) and via the respiratory system to the systemic circulation [66,67]. Phthalates can pass from maternal blood to the fetus. Mose et al. took five placentas from uncomplicated pregnancies in which babies were delivered via elective cesarean sections and developed a dual recirculating placenta perfusion system. Eight different phthalates were inserted into the circulation to identify whether they could pass through umbilical cord blood. They found that mMP, mEP, mBP and mEHP can be passed from the maternal to the fetal circulation [68]. Another study targeted seven genes in placental tissues using polymerase chain reaction (PCR) and examined the association between their expressions and phthalate exposure. Differences in the expressions of the genes and a change in the function of the placenta after exposure to five phthalates were found, revealing the permeability of the placenta to these chemicals [69]. Two studies showed that exposure to MEP phthalate in pregnancy reduces the age of menarche in daughters. A study in Mexico City measured the MEP concentration in urine samples from pregnant women in the third trimester and from their daughters aged 8–13 years. It was reported that gestational exposure to MEP resulted in a 2.66 times increased odds for early menarche and that exposure to high concentrations of MEP close to the time of puberty also decreased the age of menarche (OR:2.58) [16]. The prospective study by Watkins et al. is a unique study that revealed that only considering the third-trimester level of MEP does not allow for the determination of the decisive effect of exposure to a high MEP concentration at the beginning of pregnancy on early menarche in future adolescents. The authors measured the concentrations of nine phthalates in urine during the first, second and third trimesters of pregnancy and recorded the age of menarche and the level of the reproductive hormones in the girls. They found that there was 3.9 times increased odds of experiencing early menarche when there was a high concentration of MEP in the first three months of the gestational period. Moreover, exposure to several phthalates, except for MEP and MiBP, elevated testosterone levels [17]. However, a study of a cohort of farmworkers, in which fetal exposure and pubertal exposure to phthalates were measured through urine samples taken during pregnancy and the 9th year of life, respectively, reported that a high pubertal level of MEP was inversely associated with the age of menarche (1.3 month delay). Moreover, prenatal exposure to MEP lowered the age of menarche [32]. Oskar et al. studied 41 environmental chemicals of seven classes, including phthalates, phenols, phytoestrogens, PAH, parabens and metals, in blood and urine samples and obtained data on the menarche of girls who were aged 12 or above at the time of the study. Tree-based procedures were used to determine the effect of each chemical either alone or in combination on menarche. They proposed a positive association between pubertal lower levels of MEHP (<2.36 ng/m) and the onset of menarche rather than with high levels (OR: 1.36 95% CI: 1.02–1.80) [18]. A Mexican study investigated the effect of exposure to nine phthalates in pregnant women on the menarche of their daughters. The mothers provided urine samples up to three times during gestation to determine their chemical exposure. Their daughters were clinically evaluated for Tanner stage and menarche two times, at the ages of 8–14 and 9–18. The authors found that, at high concentrations of MBzP, earlier menarche had a hazard ratio of 3.86 (95% CI: 1.38, 15.5) [19]. A prospective study of 179 girls investigated the association between prenatal exposure to eight phthalates, using two maternal urine samples, and the age of menarche in their children. The girls were followed from the 9th year of life every 9 months for the recording of pubertal stage. High concentrations of MCNP, MCPP and MCOP delayed menarche (mean shifts: 2.4 months, 3.3 months and 2.1 months, respectively) and pubarche in normal-weight girls, but this was not found in girls with overweight and obesity. MBzP was inversely associated with menarche and thelarche in all girls [20]. A negative association between childhood exposure to MCPP and the age of menarche was also reported in a cohort of 1051 girls (HR = 0.73, 95% CI: 0.59–0.91) [28]. One study analyzed exposure to phthalates in two stages of development (B1 and B4 Tanner breast stages) of girls in Chile using urine samples. It was shown that ΣDEHP delayed the age of menarche (OR: 0.77, 95% CI: 0.60–0.98), while MMP decreased it (OR: 1.3 95% CI: 1.10–1.53) [30]. In a study by Hart et al. in 2014, members of the Western Australian Pregnancy Cohort Study were examined in order to evaluate maternal exposure to phthalates and the subsequent adverse effects on the reproductive systems of their daughters, particularly on the age of menarche, PCOS, several sex hormones and measurements of the uterus and ovaries. The 123 serum samples that were available from the pregnant women were stored for nearly 20 years at −80 °C and were analyzed to determine the level of phthalates in the intrauterine period. The authors published that the concentration of ΣDEHP was positively associated with early menarche, and this result was very close to statistical significance (*p* = 0.069) [21]. In a study of two cohorts established in Duisburg and Bochum in Germany, boys and girls aged 8–10 years were examined, and the concentrations of 21 phthalates were measured using urine tests. The children completed a questionnaire each year for 3 years, based on the Pubertal Development Scales. Kasper-Sonnenberg et al. found a negative association between DiDP and menarche (ORs 0.74–0.82), a 0.7–0.82 increased odds of reaching menarche after DEHP exposure and a reduction in the age of menarche at high DiNP concentrations (ORs 1.08–1.14) [22]. Interestingly, a 20-year study reported an interaction between gestational exposure to phthalates and the onset of menarche. Participants from the Raine study Gen2, which was first designed to examine the link between early life events and subsequent behavior and health, were examined. The high tertile of ΣDiNP and middle tertile of MCMHP delayed the age of menarche (OR = 0.73 and 0.71, respectively), and the middle tertile of the sum of HMW phthalates was also inversely associated with the age of menarche (OR = 0.72) [23]. In a Korean school study, 236 girls from the 7th to 9th school grades provided urine samples for the estimation of exposure to phthalates, and their parents completed a questionnaire about the age of menarche of their daughters. Early menarche was defined as before the 6th grade of school. Exposure to high logarithmic levels of MnBP resulted in a 2.09 times increased odds of menarche before the 6th grade, and the group with high logarithmic concentrations of the sum of eight phthalates had an increased likelihood of menarche of 2.22 times [24]. Zhang et al. tested the urine of 430 girls and boys for six phthalates, and the children underwent a clinical estimation of their puberty stage by doctors at the first visit and at 18 months after the first visit. The age of menarche, testicular volume, pubic hair and breast development were assessed. Regarding the age of menarche of the girls, the authors found a 70% increase in the possibility of early menarche at high concentrations of MEHHP and MEOHP [25]. In summary, the change in the age of menarche depends on the compound of phthalates and the period of exposure (intrauterine or childhood).

### 3.3. Phenols

Phenols have been used for over 50 years in the manufacturing of polycarbonate plastics (food and drink packaging) and epoxy resins (coatings of metal cans) [26]. Some representative compounds are bisphenol A (BPA), triclosan and DCPs. A study in mice found that bisphenol A has an estrogenic effect. Exposure to BPA decreased the degree of DNA methylation in the genes Igf2r and Peg3 during oocyte development, resulting in the elevation of active estrogen receptors [70]. From the 2003–2010 NHANES survey, 987 girls aged from 12 to 19 years old were assessed for BPA concentrations in their urine and questioned about their age at menarche. Early menarche was defined as menarche before the age of 12. It was shown that bisphenol A was negatively related to menarche with an OR = 0.55 (95% CI = 0.31, 0.99). Furthermore, girls with a BMI in the >85th percentile and exposed to low levels of BPA had a 1.65 times increased likelihood of early menarche compared to normal-weight girls with low levels of phenols [26]. One cross-sectional study also demonstrated retardation of the age of menarche after BPA exposure. In a population of 655 girls in China, a negative association between moderate and high urine BPA levels in the childhood period and the age of menarche was found (OR = 0.73 and 0.72, respectively). Interestingly, in girls aged 9–12, a positive relationship between BPA and stage 2 pubic hair development was found, while in girls aged 15+ years, BPA exposure was inversely associated with stage 5 pubic hair [27]. A prospective study of 179 girls also reported that high fetal exposure to BPA, examined through maternal urine, was inversely associated with the age of menarche (3.2 month delay) in normal-weight girls. Moreover, retarded pubarche was also recorded in normal-weight girls exposed to BPA [20]. Wolff et al. selected 1051 girls, measured the levels of phenols in their urine and administered a self-report questionnaire about the age of menarche. They proved that the 5th quintile of 2,5-DCP concentration had 1.34 times higher odds of early menarche versus the 1st quintile, while the 5th quintile of phenol enterolactone delayed the age of menarche by 4 months when compared to the 1st quintile [28]. Bigambo et al. chose 297 girls aged 10–19 years old from the NHANES 2013–2016 survey and studied both the individual and mixture effects of phenols, parabens and phthalates on female reproduction. Three statistical approaches were utilized, namely, BKMR, LASSO and GLM, and serum SHBG, E2 and testosterone were measured in the adolescents. The authors demonstrated that, in both the BKMR and adaptive LASSO approaches, 2,5-DCP increased estradiol levels, whereas a high concentration of 2,4 DCP was linked to menarche before 12 years of age in the GLM approach. Additionally, the sum of the chemicals was correlated with serum testosterone and SHBG but not with estradiol or the age of menarche in the BKMR model [29]. Equally interesting is that the authors analyzed 26 phenols and phthalate compounds in a cohort of girls with a low socioeconomic status. The urine concentrations of the chemicals were measured before the B1 Tanner stage and at the B4 Tanner stage. A high concentration of 2,5-dichlorophenol in early childhood increased the likelihood of early menarche by 1.13 times (CI: 1.01–1.27) compared to in girls with no exposure. Benzophenone-3 in Tanner stage B1 was related to the early onset of menarche (HR: 1.17; 95% CI: 1.06, 1.29). It was shown that these phenols decrease the age of menarche when they are present in high concentrations at the B1 Tanner stage, but at the B4 stage, there was no statistically significant difference [30]. Furthermore, another study of 440 girls measured the urine levels of phthalates, phenols and parabens. On the same day, the girls completed a questionnaire about the age of their menarche. The authors revealed a hazard ratio of 1.1 for reducing the onset of menarche with 2,5-DCP pubertal exposure (95% CI: 1.01–1.19) after adjustment for race and BMI. Moreover, a mixture of 2,5-DCP and 2,4-DCP was associated with early menarche (HR: 1.09 95% CI: 1.01–1.19) [31]. A prospective study of a farmworker population measured the concentrations of three phthalates, four phenols and methyl and propyl parabens in the urine of pregnant women and their children in the 9th year of life. It was found that a doubling of the prenatal concentrations of triclosan and 2,4 DCP was associated with the occurrence of menarche 0.7 months and 0.8 months earlier, respectively. Moreover, an association was reported between the peripubertal doubling of 2,5 DCP and later pubic hair development [32]. Ultimately, prenatal and childhood exposure to BPA were inversely associated with the age of menarche, whereas high levels of peripubertal 2,5 DCP and benzophenone-3 and high levels of prenatal and peripubertal 2,4 DCP decreased the age of menarche.

### 3.4. Organochlorines

Organochlorine chemicals consist of carbon, chlorine and other elements. Representative compounds include polychlorinated biphenyls (PCBs), dichlorodiphenyl trichloroethane (DDT) and dichlorodiphenyldichloroethylene (DDE). According to the Centers for Disease Control and Prevention (CDC), people are exposed to organochlorines mainly through nutrition, particularly by consuming contaminated meat, fish and milk products. PCBs are used as insulating materials in electrical equipment (transformers and capacitors), as plasticizers (softening materials) and for many other industrial purposes. Their usage has stopped in many countries, but they still exist in many parts of the world [38]. In a cross-sectional study by Denham et al., 138 girls aged 10–16 from the Akewsasne Mohawk Nation were examined. Blood tests were performed for several chemicals, including polychlorinated biphenyls, and the girls were questioned regarding the time of their menarche. The authors proposed that high logarithmic levels of four compounds of likely estrogenic PCBs (E-PCBs) predict earlier menarche (b = 2.13) [33]. A study of 792 girls in Belgium examined the association between peripubertal exposure to PCBs (138,153,180 congeners), using serum samples, and to lead and cadmium, using urine and serum samples, and pubertal stages. Information about pubertal stages was recorded after a clinical examination and questionnaires. It was reported that PCBs resulted in an increased likelihood of menarche after the age of 12.9 years (OR = 1.41, 95% CI: 1.07–1.86 *p* = 0.01). Moreover, higher exposure to lead was related to retarded pubarche [34]. Attfield et al. measured blood concentrations of PCBs and organochlorine pesticides (OCPs) in girls and examined the association with the age of menarche. The authors showed that, in a model adjusted for all covariates, except for BMI, the fourth quartile of the childhood concentrations of PCBs and OCPs retarded the age of menarche compared to the 1st quartile (OR = 0.67 and 0.66, respectively) [35]. DDT was first used on a large scale in 1945. Subsequently, it was used against insects in rural regions, particularly in those infected with malaria. In 1972, its use was forbidden in the US [71]. Several studies have demonstrated accelerated menarche following exposure to DDT and DDE. In a Chinese study that enrolled textile workers aged 21–24 who had no professional exposure to organochlorines, exposure to DDT was estimated using serum samples. PCBs were also measured to exclude the possibility of an effect of a mixture of chemicals. Women in the 4th quartile of concentrations of total DDT experienced menarche 1.1 years earlier than those in the 1st quartile. An elevation of 10 ng/g DDT was linked to a 0.2 year reduction in the age of menarche. The study found that the 4th quartile of total DDT caused 2.78 times increased odds of experiencing menstrual cycles of length < 21 days as opposed to the 1st quartile [36]. Of additional interest is that one study proved the effect of DDT on the menarche of the following two generations. This study was based on the 3Gs study (the Three Generations of Breast Cancer study), and the serum of the participants, which was obtained in that study and examined 40 years later. Serum was mainly taken from the grandmothers from 1 to 3 days after giving birth and, on some occasions, in the third and second gestational trimesters. o,p’-DDT, p,p’-DDT and p,p’-DDE levels were measured; the granddaughters completed a form about their age at menarche; and their BMI was calculated. Cirillo et al. showed that girls whose grandmothers had the 3rd tertile of concentration of serum o,p’-DDT had a 2.1 times increased likelihood of menarche before the 11th year of life (95% CI: 1.1–3.9). In the same study, the 3rd tertile of concentration of o,p’-DDT of the grandmothers was related to their granddaughters experiencing an increased risk of BMI > 30 (OR 2.6) [37]. A cohort of 151 girls involved in fishing was examined using blood samples to determine exposure to DDE and PCBs during the gestational period, and their daughters were approached by telephone to report their age of menarche. The authors reported that an elevation of 1 μg/dL DDE in the mothers reduced the time of menarche of their daughters by 0.07 years (P: 0.038), but after controlling for BMI, there was no statistically significant difference. In contrast, PCB was not statistically correlated with the age of menarche [38]. A prospective longitudinal study of 316 girls investigated the association between fetal and lactational exposure to DDE and PCBs and pubertal stages. Prenatal exposure was assessed through the concentration in maternal serum, while lactational exposure was assessed through the concentration in breast milk and maternal blood and the duration of breastfeeding. The authors reported that exposure to PCBs and DDT, either prenatally or during the lactation period, had no effect on the age of menarche or other pubertal stages [39]. Furthermore, a follow-up study in Denmark found no association between in utero exposure to several organochlorines (p,p’-DDE, HCB and six PCB congeners) and the age of menarche of daughters. However, the enrolled girls were referred to an ultrasound examination for the estimation of the number of ovarian follicles, and the numbers of follicles reported in those with high exposure to p,p’-DDE and HCB were 28% and 30% lower than the reference values [40]. Namulanda et al. conducted a case–control study and investigated the interaction of nine organochlorines in the intrauterine period with the age of menarche of the daughters. In total, 218 of the 3682 girls who had reached menarche before 11.5 years old and a random group of 230 girls who experienced menarche at ≥11.5 years old were selected. However, the authors did not find a relationship between prenatal exposure to organochlorines and menarche [41]. Nonetheless, only one study has found that organochlorines delay menarche. Axmon studied four different groups of people, namely, (a) 545 women from fishing families on the Swedish east coast (contaminated with organochlorines), (b) 1252 women from fishing families on the Swedish west coast (less polluted), (c) 634 women from non-fishing families on the east coast and (d) 869 women from non-fishing families on the west coast, and all of them reported their first menstruation. The authors demonstrated that the median ages of the menarche of females in the same region who ate contaminated fish from the east coast and females who did not were 13 years and 12.8 years, respectively. There was no difference in the time of menarche between groups a, b and d [42]. Overall, peripubertal exposure to PCBs was related to a retardation of the age of menarche in two studies, while one study supported earlier menarche. DDT and DDE were found to decrease the age of menarche.

### 3.5. Perfluoroalkyls and Polyfluoroalkyls

PFASs are chemicals that are used in everyday products. They are found in clothing, cookware, automobiles, electronics, fire-retarding foams, surfactants, and lubricants. A major source of PFASs is the diet. In total, 67 to 84 percent of exposure to perfluorooctanoic acid (PFOA) and 88–99% of exposure to perfluorooctyl sulfonic acid (PFOS) in humans come from food products. Furthermore, the home environment plays a crucial role in total exposure to PFASs. Home air was found to be responsible for 40% of exposure to PFOA in 25% of women who lived in environments with high dust concentrations [72]. A cohort study reported that perfluoroalkyls can pass through the placenta to the fetus. A total of 123 pairs of samples of mothers’ blood and omphalic cord blood were obtained at the Oslo University Hospital. Maternal blood was collected at the 37th week of gestation for the detection of seven PFC compounds. The level of PFASs measured in cord blood was 30–79% of that of the pregnant mother [73]. In a Danish study that was initially designed to determine the effect of diet during the gestational period on pre- and peri-natal outcomes, the authors examined the interaction of perfluoroalkyls PFOA and PFOS in pregnant women with the age of menarche of their daughters. Maternal blood samples were taken at the 30th week of gestation for the estimation of PFAS exposure, the age at menarche of 89 girls was only obtained by recall, and 254 girls were recommended for an ultrasound examination and measurements of hormones in the blood. The authors showed that girls born to mothers with high exposure to PFOA had a 5.3 month (95% confidence interval: 1.3; 9.3) retardation of menarche in comparison to girls born to mothers with a low concentration tertile. Additionally, a relationship between PFOS and the age of menarche was not found [43]. Christensen et al. used data from the United Kingdom study ALSPAC (Avon Longitudinal Study of Parents and Children) and measured eight polyfluoroalkyl chemicals (PFCs), including PFOA and PFOS, in pregnant women’s serum. The daughters provided their age of menarche via questionnaires. The study included 218 girls who reported menarche at <11.5 years age and a randomized control group of 230 girls. There was no statistically significant difference between PFCs and menarche onset [44]. A cross-sectional analysis of 2931 girls measured the serum peripubertal concentrations of PFOA and PFOS and found a delay in menarche of 130 days and 138 days with high levels of PFOA and PFOS, respectively, compared with those with low levels [45]. A prospective study investigated prenatal exposure to PFOA, PFOS, PFHxS, PFHpS, PFNA and PFDA, through maternal blood samples taken in the first gestational trimester, and followed the daughters for the assessment of puberty onset. A combined female puberty indicator was designed, which was the average of all puberty variables. Interestingly, each of the five PFASs was positively associated with the age of menarche and other puberty variables; however, compared to high exposure, medium exposure to PFOS, PFHpS and PFDA resulted in menarche occurring earlier. Furthermore, exposure to all PFASs was related to an earlier combined puberty indicator [46]. In summary, childhood exposure to PFOA and PFOS provoked delayed menarche, while high prenatal levels of PFASs were related to retarded or earlier menarche or had no effect in the above studies.

### 3.6. Metals

Cadmium and lead are the metals that humans are exposed to the most. Cadmium is encountered in the environment after mining operations and industrial processes and as a byproduct of petroleum combustion [49]. Lead has been used as both a spermicide and abortifacient and has even been implicated in the fall of the ancient Roman Empire. Wu et al., who used data from the NHANES III survey (Third National Health and Nutrition Examination Survey), noticed that lead increases the age of menarche. The enrolled girls underwent a clinical examination to determine Tanner breast stage and pubic hair development, and blood was collected to estimate peripubertal exposure to lead. Three lead levels were determined: low (0.7–2 μg/dL), medium (2.1–4.9 μg/dL) and high (5–21.7 μg/dL). The percentages of girls who experienced menarche at 12 years of age comprised 68.3% of those with low exposure, 44.3% of those with medium exposure and 38.5% of those with high exposure to lead. At 10 years of age, 90 out of 125 girls with low exposure, 82 with medium exposure and 36 with high exposure to lead had already attained stage 2 pubic hair development. No relationship was found between breast development and lead concentration [47]. Two cross-sectional studies indicated a delay in the age of menarche after lead exposure [33,48]. One of these studies enrolled 2186 girls and analyzed the association between lead serum concentration and puberty stages. The age of menarche was also recorded. The participants comprised 600 white girls, 805 African American girls and 781 Mexican American girls. The authors showed that, in African American and Mexican American girls, blood lead concentrations of 3 µg per deciliter were inversely associated with the age of menarche (3.6 month retardation) and breast and pubic hair stages. However, in white girls, lead was not related to puberty onset [48]. Cadmium affects the hormonal profile of women. It was reported that cadmium interacts with regions of alpha estrogen receptors (C381, C447, E523, H524 and D538), activating them and producing an estrogenic effect [74]. Cadmium can be transported from the ground to agricultural products. Exposure can occur through the diet or cigarette smoking. Reynolds et al. published a study based on data from the Growth and Lifestyle Study (GRLS), which was first conducted to show the effect of isoflavones on puberty. They selected 211 girls aged 10–13 years old, obtained monthly responses about the occurrence of first menstruation and conducted yearly interviews in which the girls self-estimated their breast and pubic hair development. The participants also collected overnight urine samples, and cadmium levels were measured. It was found that girls with a cadmium concentration <0.2 μg/L had 58% increased odds of reaching early menarche compared to those with a concentration of ≥0.4 μg/L after adjustment for race, creatinine concentration, body fat percentage and height. Cadmium concentration was negatively associated with pubic hair growth (OR = 0.21), but there was no correlation between cadmium concentration and Tanner breast stage [49]. Chen et al. included three different areas in their study: the first was heavily polluted by cadmium, the second was moderately polluted, and the third was the control. Cadmium exposure was measured via the concentration of the chemical in rice. A total of 137 people from the control area participated, and a total of 292 from the areas exposed to cadmium participated. The effects of cadmium on menarche and menopause were investigated and a total of 223 girls reported their age of menarche. The median age of menarche was 15 years in the control population, whereas it was 14 years in the population with moderate and high cadmium concentrations. Furthermore, people from the heavily polluted areas had a 3.7 times increased odds of experiencing menarche before the 13th year of life compared to the control group. The population with a moderate concentration of cadmium had an OR of 1.3 for menarche before 13 years of age [50]. A prospective mother–child study in Bangladesh examined prenatal and pubertal exposure to cadmium, lead and arsenic and the relationship with the age of menarche in 935 girls. Prenatal exposure to metals was assessed through the concentration in maternal erythrocytes, while pubertal exposure was assessed through urine samples from the girls at 5 and 10 years of age. A high cadmium concentration at 5 (HR = 0.8, 95% CI: 0.62–1.01) and 10 years of age (HR = 0.77, 95% CI: 0.60, 0.98) delayed menarche compared to a low concentration. Furthermore, a high quartile of urinary lead concentration at 10 years of age lowered menarche (HR = 1.23, 95% CI: 0.97, 1.56), while a high arsenic concentration in maternal erythrocytes was associated with retarded menarche (HR = 0.79, 95% CI: 0.62, 0.99) [51]. A study in a Mexican cohort measured the concentrations of several metals (Al, As, Ba, Cd, Co, Cu, iron, Mn, Mo, Sb, Ni, Se and Zn) in urine samples from pregnant women during the third gestational trimester and their daughters at 8–13 years of age. The pubertal stages and onset of menarche of the female children were recorded by pediatricians, and a blood sample was acquired to assess the levels of reproductive hormones during the early-teen visit. A positive association between high exposure to Ba, Al and Co and earlier menarche was reported, while high peripubertal cadmium exposure delayed the age of menarche (OR = 0.69 95% CI: 0.48–1). Of additional interest is that peripubertal Co was associated with earlier menarche (OR = 3.8), and in utero Mn was related to later menarche (OR = 0.29) [52]. In a case–control study, the correlation between the age of menarche and arsenic exposure was assessed. A total of 280 girls living in four Indian villages (A1, A2, A3 and A4) with water polluted by arsenic and 70 living in a non-polluted village (C1) serving as a control group were included. The authors showed a negative correlation between the age of menarche and arsenic exposure. The median ages of menarche were 12.72, 12.8, 11.96 and 12.5 in the A1, A2, A3 and A4 villages, respectively, while it was 11.76 in the C1 village [53]. In summary, childhood exposure to lead was associated with a retardation of the age of menarche in two studies while in one study earlier menarche was mentioned. High antenatal levels of cadmium were negatively related to the age of menarche in three studies; in contrast, in one study, earlier menarche was shown.

### 3.7. Air Pollutants

In the atmosphere, dioxins and dioxin-like compounds may intervene in the female reproductive system. They include dibenzodioxins, divenzofurans, PBBs and hydrocarbons. Chiu-Yueh Yang et al. studied people called Yucheng, from the Chinese word meaning oil illness, who were exposed to PCBs and divenzofurans (PCDFs) through polluted cooking oil. Twenty-four years after exposure, 445 women (exposed and control groups) were asked about their menstrual cycles by telephone. The authors reported slightly earlier menarche in Yucheng women with skin lesions (*p*-value = 0.08). Additionally, the exposed population displayed a decrease in cycle length by 0.7 days (95% CI: −1.4–0.0, *p* = 0.04) and an increase in menstruation length by 0.5 days compared to the control group [54]. In 1999, 120 Belgian girls were enrolled in a study estimating the serum concentration of the sum of four PCB congeners and the sum of all dioxin-like compounds (with the use of the Calux biodetection system). The girls were living in either a rural Belgian village used as a control, an urban village that was close to two waste incinerators or another urban village that hosted a nonferrous smelter. The age of menarche and the stage of puberty were evaluated by physicians. There was no association found between dioxins and the age of menarche, while a retardation of Tanner breast stage was found [55]. A retrospective study examined the effects of 2,3,7,8-tetrachlorodibenzo-p-dioxin (TCDD) on the onset of menarche. In 1976, a chemical explosion occurred in Italy, and females living in nearby regions, who had not yet reached menarche, were examined for the presence of TCDD in their serum. After many years, the age of menarche of these women was reported. It was demonstrated that a 10-fold increase in TCDD concentration was not related to the age of menarche (HR = 0.95, CI: 0.83–1.09) [56]. Blanck et al. conducted a study where they examined the effect of PBB and PCB exposure on the age of menarche, following an industrial accident in Michigan in 1973, resulting in food contamination. Serum from pregnant women was analyzed to estimate exposure to these chemicals. An electronic questionnaire was sent by email, asking for information on the age of menarche and the lactation period of daughters born from these women. It was found that girls exposed to a high concentration of PBBs during the gestational and lactation periods had a median age of menarche of 11.6 years, while those who were not breastfed had menarche at 12.69 years. Adolescents exposed to low levels of PBBs in utero and through breastfeeding had menarche at 12.2–12.7 years. A positive correlation between PBBs and menarche was observed, even after adjustment for maternal ages at menarche and at birth, PCB level, smoking frequency and economic status. The daughters who were also breastfed and were exposed to high levels of PBB intrauterinely had a 3.4 times increased odds of early menarche compared to those who were exposed to low levels only in utero [57]. Particulate matter (PM) is composed of diverse components, including endocrine-disrupting chemicals (EDCs), heavy metals and polycyclic aromatic hydrocarbons. A survey that used data from the 5th KNHANES study selected 639 Korean girls aged 13–17 years old and reported the effects of 1-year, 2-year and 3-year averages of annual concentration of PM10 on the age of menarche. PM10 was measured hourly in the air to calculate annual exposure. It was found that a 1 μg/m^3^ rise in 1-year, 2-year and 3-year annual PM10 concentrations led to a decline in the age of menarche of 0.046 years, 0.038 years and 0.031 years, respectively, after adjustment for BMI, the maternal age of menarche, smoke exposure from the family environment, town population and economic status. In addition, it was reported that, when the time of exposure to PM10 was shorter, there was a stronger correlation with menarche. An elevation of 1 μg/m^3^ of PM10 in 1 year was linked to a 1.08 times increased odds of experiencing early menarche (95CI:1.08–1.12), while in 3 years, the OR was 1.05 [58]. A recent study by Wronka et al. investigated the effect of several air pollutants on the age of menarche. In this study, 1257 female university students in Poland were enrolled, and data about air PM10, PM2.5, sulfur dioxide (SO_2_), nitric oxide (NO) and benzene (C_6_H_6_) in several regions were obtained from air pollution stations. The pollution level was divided into three classes: low, medium and high. The girls completed a questionnaire about their age of menarche and social and economic features. The authors demonstrated that, in class 3 PM10, the median age of menarche was 12.54, while in class 1, it was increased (13.32 years). An elevated concentration of PM2.5 was also associated with a decreased age of menarche. At low air pollution levels of NO, the age of menarche in girls was 12.98, while in heavily polluted areas, it was 12.62. A 3-fold increased likelihood of menarche before the 11 years of age was found in girls living in heavily polluted regions compared to those with low exposure. PM2.5 was also linked with a 3.25 times increased odds of early menarche in the high pollution group than in the low pollution population (95% CI: 2.34–4.8). Of additional interest is that, when the chemicals were calculated as a mixture, an increase in their concentration was correlated with a decrease in menarche, after adjusting for family socioeconomic status [59]. A prospective study of 358 girls in San Francisco examined the exposure to polycyclic aromatic hydrocarbons (PAHs) and the effect on the age of menarche. Every six months, the girls provided a urine sample for the estimation of PAH concentrations and the age of menarche, and pubertal stages were determined through questionnaires, completed by the mothers or the girls. No association between PAH exposure and the age of menarche was found. However, it was shown that exposure to high levels of 2-NAP,1-PHEN and summed hydroxy phenanthrenes in girls with overweight resulted in a two times increased odds of faster breast development compared to normal-weight girls with low exposure. Furthermore, a positive relationship between 1-NAP and early pubarche was reported [60]. Furthermore, Kehm et al. analyzed the correlation between prenatal exposure to PAHs, puberty stage and body composition. The concentrations of high-molecular-weight PAHs were assessed using backpack air monitors, while low-molecular-weight pyrenes were assessed using umbilical cord blood samples. The pregnant women carried the backpack for 2 days in the third gestational trimester. The authors reported that high exposure to the sum of the eight HMWs retarded menarche by 0.59 years (95% CI = 0.06, 1.11). The higher pyrene concentrations in umbilical cord blood was inversely related to breast development and growth spurt onset [61]. Overall, pubertal exposure to PM10, PM2.5 and NO decreased the age of menarche while TCDD had no effect. Intrauterine high levels of PAHs increased the age of menarche, whereas pubertal high levels had no effect. PBB exposure prenatally and through lactation decreased the age of menarche.

### 3.8. Polybrominated Diphenyl Ethers

PBDEs are found in furniture fabric, televisions, personal computers and wire coverings, where they are used to decrease the possibility of ignition and to slow the burn rate if the products catch fire. People can be exposed via air, dust or nutrition. Furthermore, PBDEs can be passed from a mother to her baby either transplacentally during intrauterine life or through lactation during antenatal life [75]. Their structure consists of bromine and two phenol rings. Different positions of bromine atoms result in another compound, 209 of which are known. It has been shown that PBDEs act as agonists (lower-molecular-weight compounds up to hexa-PBDEs) or antagonists (hepta-PBDEs and higher and hydroxylated PBDEs) of estrogen receptors and antagonists of progesterone and androgen receptors [76,77,78]. In a study by Chen et al., which included 271 girls aged 12–19 years from the NHANES 2002–2004 study, the effect of PBDEs on the age of menarche was assessed. From a serum sample collected after menarche, exposure to BDE-28, -47, -99, -100, -153 and -154 was estimated and adjusted for lipids. The girls were questioned about the onset of menarche. In general, each of the six PBDEs was associated with a decrease in the age of menarche at high concentrations. In particular, BDE-47 in the 4th quartile of concentration declined the age of menarche by 0.49 years compared to the 1st quartile (*p* < 0.05). Each natural log unit of total BDEs was correlated to a 0.16-year drop in the age of menarche and a 1.76 times increased likelihood of reaching menarche before 12 years of age when sex and BMI were included in the analysis [62]. Attfield et al. published a study using data from the Breast Cancer and Environment Research Program in California and Ohio and examined the consequences for menarche after exposure to PBDEs, organochlorines and PCBs. Visits were held each year to take menarche and anthropometric measurements, and blood samples were obtained for the measurement of the chemicals’ concentrations. As far as PBDEs were concerned, the 4th quartile level was related to a delay in menarche compared to the 1st quartile (OR = 0.75, 95% CI: 0.58–0.97) after adjustment for ethnicity, household income, maternal education and age at birth. When BMI was considered, no statistically significant difference was observed [35]. Finally, Harley et al. analyzed four PBDEs (BDE-47, -99, -100 and -153) in mothers’ blood during pregnancy or at birth and in their child’s blood at the 9th year of life. The participants were acquired from the Chamacos study, which enrolled women from Mexican families, in order to evaluate the effect of the environment on child development. Tanner stages were evaluated every 9 months, and the onset of menarche was mentioned. The authors showed a 2-fold increased likelihood for later menarche when there was a high concentration of the sum of PBDEs in the intrauterine period (95% CI: 0.3–0.8 for early menarche). However, peripubertal exposure to a mixture of PBDEs was not related to the timing of menarche. In addition, individually, there was no relationship, except for the case of BDE-153, in which each 10-fold increase at the age of 9 years old was linked to a 7.2 month later age of onset of menarche [63]. Eventually, gestational exposure to PBDEs retarded the age of menarche but a high pubertal concentration of PBDEs was found to decline or delay or had no effect on the age menarche.

### 3.9. Mixture of Chemicals

Humans are exposed to many endocrine-disrupting chemicals throughout their lives. For this reason, a very interesting study investigated the effect of fetal exposure to a mixture of three chemical classes (PFAS, PCBs and organochlorines), through examining maternal serum during pregnancy, on the age of menarche. It was reported that the mixture of EDCs was not related to the age of menarche. Using weighted quantile sum (WQS) regression, a one-decile increase in the levels of the mixture of the three chemical classes was found to be associated with an odds ratio of 0.89 (95% CI: 0.76, 1.05), and when using BKMR in the 60th percentile of the concentration of the chemical mixture compared to the median, the OR for early menarche was 0.98 (95% CI: 0.91, 1.05) [64].

## 4. Conclusions

**Figure 2:**
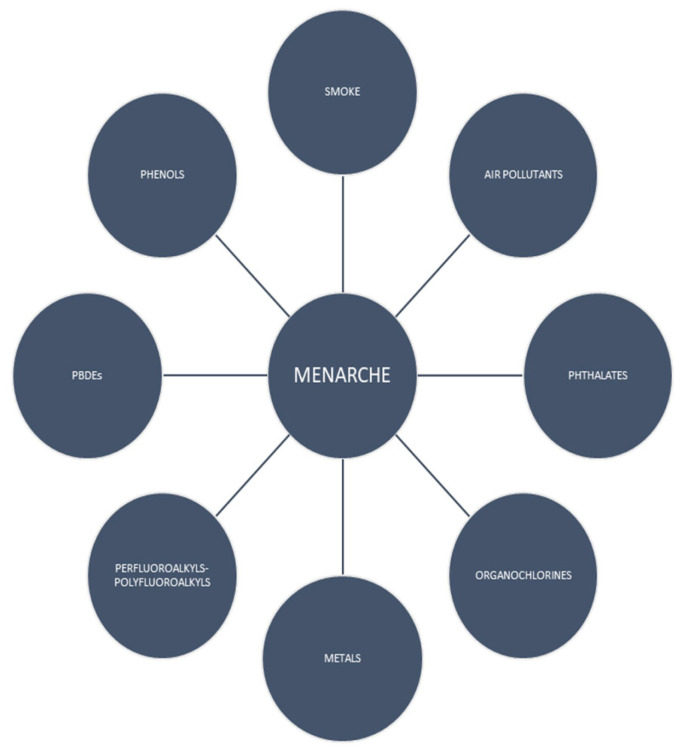
Chemical factors that affect menarche.

This is a comprehensive literature review of all the evidence regarding the impact of chemicals on the age of menarche (Figure 2). Τhe effects of chemicals on menarche are dependent on the level and duration of exposure. Intrauterine exposure sometimes has a different impact from antenatal exposure. High concentrations of chemicals do not usually affect the female reproductive system in the immediate future, but the consequences come to light after many years, even in the following generations. A reduction in the menarche of offspring increases the risks of cancer, obesity and diabetes. Currently, the rapid progress in technology and improvements in lifestyle are satisfactory. However, the period spent for estimating the effect of chemicals on the age of menarche is short and new products are manufactured every day; thus, further studies should be conducted in order to evaluate the significance of these chemicals on the timing of menarche to improve female health. In addition, society should consider reducing the amount of chemicals released into the environment.

## Data Availability

Data used in this study are presented within the manuscript.

## Supplementary Materials

The following supporting information can be downloaded at: https://www.mdpi.com/article/10.3390/children10071234/s1, Table S1: Included studies.
