# Supplementary material for: Impact of Chemicals on the Age of Menarche: A Literature Review
Source: Children (Basel). 2023 Jul 17;10(7):1234. doi: 10.3390/children10071234 (PMC10378553; doi:10.3390/children10071234)
Supplement: Supplementary file 1 [file children-10-01234-s001.zip › children-2476279-supplementary.pdf]

Table S1. Included Studies.

First Author (Year)	Design	Sample Size (Girls)	Median Age of Participants	Test Specimen	Metabolites	Time of Exposure	Menarche Associated with High Exposure	Further Findings Associated with High Exposure
Zhang (2015) [8]	Retrospective	751	12,87	Unapplicable	Smoke	Prenatal	Earlier (OR=1,84)	Shorter cycle length
Behie (2015) [9]	Prospective	1493	12,93	Unapplicable	Smoke	Prenatal	Smoke: earlier menarche (OR=1,4)<br>Older maternal age of menarche and higher birth weight: delayed menarche<br>Higher BMI at age 8-9: earlier menarche	None
Houghton (2018) [10]	Prospective	1090	39-49	Unapplicable	Smoke	Prenatal	Earlier menarche by 0,56 years in rapid growth<br>Delayed by 0,53 years in slow growth	None
Windham (2004) [11]	Prospective	994	16-17	Unapplicable	Smoke	Prenatal and antenatal	Prenatal and childhood exposure: earlier menarche by 4 months<br>Childhood exposure to 8+ pack years: -0,15 years	None

							Prenatal exposure to 20 cigarettes or more: -0,22 years and in non-white people -0,52 years	
Yermachenko (2015) [12]	Meta-analysis of 17 studies (prospective and cross-sectional)	Continuous variables: 92572 Categorical variables: 35855	8-98	Unapplicable	Smoke	Prenatal	Categorical variables: 15% increased odds for earlier menarche Continuous variables: -0,092 years earlier menarche	None
Kang (2020) [13]	Cross-sectional	63618	12-18	Unapplicable	Smoke	Antenatal	Secondhand smoke: earlier menarche (OR=1,12) Girls who smoked before 12 years old: earlier menarche (OR=1,68)	None
Yang (2015) [14]	Cross-sectional	20061	50+	Unapplicable	Smoke	Antenatal	Secondhand smoke from or more people: 0,38 years earlier menarche Secondhand smoke 5 days or more/week: 0,19 years	None

						earlier menarche	
Ferris (2010) [15]	Cross-sectional	262	41,8	Unapplicable	Smoke	Prenatal and antenatal	Prenatal heavy exposure: delayed menarche (OR=2,1) Childhood exposure: delayed menarche (OR=2,1) Both prenatal and childhood exposure: delayed menarche (OR=2,2)	None
Watkins (2014) [16]	Prospective	132	8-13	Urine	MEHP, MEHHP, MEOHP, MECPP, MBzP, MnBP, MiBP, MCPP, MEP	Prenatal and antenatal	MEP: earlier menarche with prenatal exposure (OR=2,66) and pubertal exposure (OR=2,58)	In utero MEHP: 5,3 times earlier pubarche and ↑ DHEAS
Watkins (2017) [17]	Prospective	120	8-13	Urine	MEHP, MEHHP, MEOHP, MECPP, MBzP, MBP, MiBP, MCPP, MEP	Prenatal	MEP in 1st trimester: 3,9 times higher odds of earlier menarche	DEHP: delayed breast development Several phthalates, except for MEP and MiBP: ↑ TT
Oskar (2016) [18]	Cross-sectional	229	14,5	Serum and urine	41 biomarkers including phthalates, phenols, phytoestrogens,	Antenatal	Lower MEHP: earlier menarche (OR=1,36)	None

					parabens, PAHs and metals		Combinations of lower MEHP with benzophenone-3, 2,4 DCP and BPA: earlier menarche	
Cathey (2020) [19]	Prospective	103	First visit: 9,98 Second visit:13,3	Urine	MEP, MnBP, MiBP, MBzP, MCPP, MEHP, MEHHP, MEOHP, MECPP	Prenatal	MBzP: earlier menarche (OR=3,86)	MBzP: increased odds for higher Tanner breast stage at 8-14 years (OR=4,62) but slower breast development over the follow-up period (OR=0,65)<br>ΣDEHP: increased odds for higher Tanner breast stage at first visit but reduced tempo for breast development over the study period
Berger (2018) [20]	Prospective	179	9-13	Urine	8 phthalates (MBzP, MCNP, MCOP, MCPP, MEHP, MEHHP, MEOHP, MECPP) and BPA	Prenatal	MCNP, MCPP, MCOP and BPA in normal-weight girls delayed menarche (2,4 months, 3,3 months, 2,1 months, 3,2 months, respectively)<br>MBzP in all girls delayed menarche by 0,7 months	MCNP, MCPP, MCOP and BPA in normal-weight girls delayed pubarche<br>MBzP in all girls delayed thelarche
Hart (2014) [21]	Prospective	121	15,1	Serum	11 phthalate metabolites	Prenatal	ΣDEHP: earlier menarche by 0,6 years	MCiOP: positively associated with uterine volume<br>MEP and the sum of all phthalates: low prevalence of PCOS and<br>MEP: negatively associated with PCO and serum AMH

Kasper-Sonnenberg (2017) [22]	Prospective	198	8,7 at first follow-up	Urine	DEHP, DiNP, DiDP, DiBP, DnBP, MEP, MBzP, MMP	Antenatal	DiDP: delayed menarche (OR=0,74-0,82 DEHP: delayed menarche (OR=0,7-0,82) DiNP: earlier menarche (OR=1,08-1,14)	None
Berman (2021) [23]	Prospective	462	20,1	Serum	32 metabolites of 15 phthalate diesters	Prenatal	Upper tertile of ΣDiNP: delayed menarche (OR=0,73) Middle tertile of MCMHP: delayed menarche (OR=0,71) Middle tertile of the sum of HMW phthalates: delayed menarche (OR=0,72)	None
Park (2021) [24]	Cross-sectional	236	7th–9th grade of school	Urine	MEHHP, MEOHP, MECPP, MnBP, MBzP, MCOP, MCNP, MCPP	Antenatal	MnBP: earlier menarche (OR=2,09) Sum of phthalates: earlier menarche (OR=2,22)	None
Zhang (2015) [25]	Cross-sectional	201	9,7±2,2	Urine	MnBP, MMP, MEP, MEHP, MEHHP, MEOHP	Antenatal	MEHHP and MEOHP: 70% increased odds	MnBP, MMP, MEP, MEHP: 4-10 times greater breast development

							for earlier menarche	
McGuinn (2015) [26]	Cross-sectional	987	12-19	Urine	BPA	Antenatal	High BPA: delayed menarche (OR=0,55) Overweight girls with low BPA: 1,65 increased odds for early menarche compared to normal weight	None
Miao (2017) [27]	Cross-sectional	655	9-18	Urine	BPA	Pubertal	Moderate BPA: delayed (OR=0,73) High BPA: delayed (OR=0,72)	Girls 9-12 years old: higher odds of pubic hair stage 2 Girls 15+ years old: lower odds of reaching pubic hair stage 5
Wolff (2017) [28]	Prospective	1051	6-8	Urine	Phenols (e.g., 2,5 DCP, enterolactone), phthalates (MEP, MBP, MiBP, MEHP, MEOHP, MEHHP, MECPP, MBzP, MCPP)	Antenatal	2,5 DCP: earlier menarche (HR=1,34) Enterolactone: delayed menarche (HR=0,82) MCPP: delayed menarche (HR=0,73)	None
Bigambo (2022) [29]	Cross-sectional	297	12-19	Urine	Phenols (2,5 DCP, 2,4 DCP), parabens, phthalates in BKMR, LASSO and	Antenatal	In GLM: 2,4 DCP: earlier menarche (OR=1,79)	In BKMR: Mixture of chemicals: positively associated with SHBG and negatively with TT In LASSO: 2,5DCP, MCOP, MBzP: positively associated with E2

					GLM approaches		MCNP: earlier menarche (OR=1,83) Mixture of chemicals: no effect	MEP, MiBP, MEOHP: negatively associated with E2 BPA and MBzP: positively associated with SHBG MCNP, MECPP: negatively associated with SHBG In GLM: BZP and MECPP negatively associated with TT
Binder (2018) [30]	Prospective	200	B1 Tanner stage: 7,9 B4 Tanner stage: 11,2	Urine	2,5 DCP, benzophenone-3, phthalates (MBP, MBzP, MCNP, MCOP, MCPP, MECPP, MEHP, MEHHP, MEOHP, MEP)	Antenatal	2,5 DCP: 1,13 increased odds for earlier menarche at B1 Tanner stage Benzophenone-3: 1,17 increased odds for earlier menarche at B1 Tanner stage MBzP, MEHP, MEHHP and MEOHP: delayed menarche (OR=0,77) MMP: earlier menarche (OR=1,3)	None
Buttke (2012) [31]	Cross-sectional	440	12-16	Urine	BPA, triclosan,benzo phenono-3, parabens, 2,4-DCP, 2,5-DCP, phthalates	Antenatal	2,5 DCP: earlier menarche (OR=1,1 Sum of 2,5 DCP and 2,4 DCP: earlier menarche (OR=1,09) Total parabens, bisphenol A,	None

							triclosan, benzophenone-3, total phthalates, and 2,4-DCP: no effect on menarche	
Harley (2019) [32]	Prospective	179	9-13	Urine	3 phthalates (MEP, MBP, MiBP), 4 phenols, methyl and propyl-paraben	Prenatal and peripubertal	Prenatal triclosan: earlier menarche by 0,7 months<br>Prenatal 2,4 DCP: earlier menarche by 0,8 months<br>Peripubertal methyl paraben: earlier menarche by 0,9 months<br>Peripubertal propyl paraben: earlier menarche by 0,4 months<br>Peripubertal MEP: delayed menarche by 1,3 months	Prenatal MEP: earlier pubic hair development<br>Peripubertal methyl paraben: earlier breast and pubic hair development<br>Peripubertal 2,5 DCP: delayed pubic hair development
Denham (2005) [33]	Cross-sectional	138	12,9	Serum	p,'p-DDE, HCB, PCBs, lead, mirex, mercury	Antenatal	Logarithmic lead: delayed menarche (β=-1,29)<br>4 E-PCBs: earlier	None

							menarche (β=2,13) p,p-DDE, HCB, mirex and mercury: no effect on menarche	
Den Hond (2011) [34]	Cross-sectional	792	14,9	Serum and urine	PCBs (138, 153, 180), HCB, p,p'-DDE, lead, cadmium	Pubertal	PCBs: delayed menarche (OR=1,41 for menarche after 12,9 years old)	Lead: delayed pubarche
Attfield (2019) [35]	Prospective	556	7,8	Serum	PCBs, organochlorine pesticides (OCPs), PBDEs	Antenatal	PCBs: delayed menarche (12,7 median age at 4th quartile vs. 11,9 in the 1st quartile) (unadjusted) Organochlorine pesticides: delayed menarche (12,4 years in 4th quartile vs. 12,1 in 1st quartile) (unadjusted) When adjusted for all, except for BMI: Q4 vs. Q1 delayed menarche (PCBs OR=0,67, OCPs OR=0,66, PBDEs OR=0,75)	None

							When BMI considered: no effect on menarche	
Ouyang (2005) [36]	Cross-sectional	446	24,9	Serum	DDT	Antenatal	4th quartile of DDT: earlier menarche by 1,1 years	4th quartile of DDT: short cycle
Cirillo (2021) [37]	Prospective	235	26	Serum	o,p'-DDT, p,p-DDT, p,p-DDE	From grandmother exposure	F0 o,p-DDT: F2 earlier menarche (OR=2,1) (without BMI considered) p,p-DDT and p,p-DDE: no effect	F0 o,p-DDT: F2 obesity among normal weight F0
Vasiliu (2004) [38]	Prospective	151	20-50	Serum	DDE, PCBs	Prenatal	Elevation of 15 μg/l DDE: earlier menarche by 1 year After adjustment for BMI: no statistically important effect PCBs: no effect	None
Gladen (2000) [39]	Prospective	316	10-15 years at first contact	Maternal serum and breast milk	DDE PCBs	Prenatal and lactational	No effect on age at menarche	No effect on pubertal stages
Kristensen (2016) [40]	Prospective	341	19,6 years	Serum	p,p'-DDE, HCB and six PCB congeners	Prenatal	No effect on menarche	p,p'-DDE: 28% lower follicle number HCB: 30% lower follicle number HCB: ↓free androgen index among non-users of hormonal contraceptives
Namulanda (2016) [41]	Case–control	448	8-17	Serum	9 organochlorines	Prenatal	No effect on menarche	None

Axmon (2006)[42]	Cross-sectional	3300	20-54	Region known to be polluted	Organochlorines	Antenatal	Exposed women had menarche delayed by 0,2 years compared to non-exposed women from the same region	None
Kristensen (2013) [43]	Prospective	343	19,6	Serum	PFOA, PFOS	Prenatal	PFOA: delayed menarche by 5,3 months<br>PFOS: no effect	None
Christensen (2011) [44]	Case–control	448	8-13	Serum	8 PFCs, including PFOA and PFOS	Prenatal	No effect	None
Lopez-Espinosa (2011) [45]	Cross-sectional	2931	8-18	Serum	PFOA, PFOS	Antenatal	PFOA: delayed by 130 days<br>PFOS: delayed by 138 days	None
Ernst (2019) [46]	Prospective	576	11 at first visit	Serum	PFOS, PFHxS, PFHpS, PFNA, PFDA	Prenatal	All: earlier menarche<br>PFOS, PFHpS and PFDA: earlier menarche in the middle vs. high exposure levels	Earlier individual puberty milestones and earlier combined female puberty indicator
Wu (2003) [47]	Cross-sectional	1235	13,5	Serum	Lead	Prenatal	Delayed	Delayed pubic hair stage<br>No effect on breast development
Selevan (2003) [48]	Cross-sectional	2186	13,4	Serum	Lead	Antenatal	Delayed by 3,6 months	Delayed breast and pubic hair stage
Reynolds (2020) [49]	Prospective	211	10-13	Urine	Cadmium	Antenatal	Delayed menarche (OR=0,42)	Delayed pubarche<br>No effect on breast development
Chen (2017) [50]	Retrospective	223	<47	Rice concentration	Cadmium	Antenatal	Heavy exposure: earlier	

							menarche by 1 year and odds = 3,7 for menarche <13 years<br>For moderate exposure: odds = 1,3 for menarche <13 years	
Malin Igra (2023) [51]	Prospective	935	13,3	Maternal concentration of erythrocytes<br>Girl's urine	Cadmium<br>Lead<br>Arsenic	Prenatal and antenatal	Cadmium concentration at 5th year of age (HR=0,8) and at 10th year of age (HR=0,77) delayed menarche<br>Lead concentration at 10th year of age decreased menarche (HR=1,23)<br>Maternal cadmium and lead concentration had no effect<br>Lead concentration at 5th year of age had no effect on menarche<br>Arsenic maternal concentration delayed	None

							menarche (HR=0,79) Arsenic concentration in girls' urine had no effect	
Ashrap (2019) [52]	Prospective	132	8-13	Urine	Al, As, Ba, Cd, Co, Cu, iron, Mn, Mo, Sb, Ni, Se, Zn	Prenatal and antenatal	Peripubertal Ba and Al: earlier menarche Peripubertal Co: earlier menarche (OR=3,8) Peripubertal Cd: delayed menarche (OR=0,69) Prenatal Mn: delayed menarche (OR=0,29)	Prenatal Zn: ↑inhibin B Pubertal Ni: ↑TT Pubertal Cu: ↓TT Prenatal Ni, Al, Cd: delayed breast development Peripubertal Al and Ba: earlier pubarche
Sen (2007) [53]	Case–control	350	Not reported	Water (tubewells)	Arsenic	Antenatal	Delayed the age of menarche at A1, A2, A3, A4 polluted villages: 12,72, 12,8, 11,96 and 12,5, respectively) At C1 control village: 11,76	None
Yang (2011) [54]	Case–control	445	Control: 38,9 Yucheng: 38,8	Region contaminated by divenzofurans and PCBs	Divenzofurans PCBs	Antenatal	Earlier in Yucheng people with skin lesions	Exposed Yucheng people had 0,7 day shorter cycle and 0,5 day longer menstrual flow

Den Hond (2002) [55]	Cross-sectional	120	17,4 ± 0,8	Serum	PCBs Dioxin (Calux)	Pubertal	Dioxin had no effect PCBs had no effect	Dioxin delayed breast development Dioxin had no effect on pubic hair PCBs had no effect on pubertal stages
Warner (2004) [56]	Retrospective	282	6,9	Serum	TCDD	Antenatal	No effect	None
Blanck (2000) [57]	Cross-sectional	327	5-24	Serum	PBBs	Prenatal and antenatal (lactation)	Breastfed girls exposed to high PBBs: earlier menarche (11,6 vs. 12,2-12,6 exposed to low levels vs. 12,7 girls who were not breastfed)	Earlier pubarche No effect on breast development
Jung (2018) [58]	Cross-sectional	639	13-17	Air levels	PM10	Antenatal	Each 1 µg/m3 increase in PM10 at 1, 2, and 3years old:earlier menarche by 0,046, 0,038 and 0,031 years respectively	None
Wronka (2022) [59]	Cross-sectional	1257	19-25	Air levels	PM10, PM2,5, NO, SO2 and benzene	Antenatal	PM10: earlier menarche (12,54 and 13,32 class 3 vs. class 1) PM2,5: earlier menarche (12,55 and 13,18 class 3 vs. class 1) NO: earlier menarche (12,62 and	None

							12,98 class 3 vs. class 1)	
John (2022) [60]	Prospective	358	6-16	Urine	PAHs (1-NAP, 2-NAP, 2- and 3-FLU, 1-PHEN, 2- and 3-PHEN, 4-PHEN, 1-PYR)	Pubertal	No effect	Girls with overweight and high exposure to 2-NAP,1-PHEN and summed hydroxy phenanthrenes: Two times higher odds of earlier breast development 1-NAP: two times higher odds of earlier pubarche
Kehm (2021) [61]	Prospective	196	11-20	Backpack monitoring of air and umbilical cord blood	Eight higher-molecular-weight PAHs and a low-molecular-weight PAH (pyrene)	Prenatal	S8 PAH: delayed by 0,59 years	S8 PAH: delayed breast development and growth spurt onset Pyrene: delayed breast development and growth spurt onset
Chen (2011) [62]	Cross-sectional	271	12-19	Serum	BDE-28, -47, -99, -100, -153, -154	Antenatal	Total PBDEs: earlier menarche by 0,1 years	None
Harley (2017) [63]	Prospective	314	9-13	Serum	BDE-47, -99, -100, -153	Prenatal and antenatal	In utero PBDEs: delayed menarche (95% CI:0.3-0,8) Childhood PBDEs (-47, -99, -100): no effect Childhood PBDE-153: delayed by 7,2 months (10-fold increase)	In utero PBDEs: no effect on pubarche and breast development Childhood PBDEs: no effect on pubarche and breast development
Marks (2021) [64]	Case–control	448	8-17	Blood	Mixture of PFAS, PCBs	Prenatal	No effect	None

and<br>organochlorines

Abbreviations: PCB, polychlorinated biphenyl; PBDE, polybrominated diphenyl ethers; PBB, polybrominated biphenyl; PM, particulate matter; BPA, bisphenol A; DCP, dichlorophenol; EDC, endocrine-disrupting compound; PHA, polycyclic aromatic hydrocarbon; HMW, high molecular weight; MEHP, mono-2-ethylhexyl phthalate; MEHHP, mono-2-ethyl-5-hydroxyhexyl phthalate; MEOHP, mono-2-ethyl-5-oxohexyl phthalate; MECPP, mono-2-ethyl-5-carboxypentyl phthalate; MBzP, monobenzyl phthalate; MnBP, mono-n-butyl phthalate; MiBP, mono-isobutyl phthalate; MCPP, mono-3-carboxypropyl phthalate; MEP, monoethyl phthalate; MCOP, monocarboxyoctyl phthalate; MCNP, monocarboxynonyl phthalate; DEHP, di-(2-ethylhexyl) phthalate; DiNP, di-isononyl phthalate; DiDP, di-isodecyl phthalate; DiBP, di-isobutyl phthalate; DnBP, di-n-butyl phthalate; MMP, mono-methyl phthalate; SHBG, sex hormone-binding globulin; TT, testosterone; DDE, dichlorodiphenyldichloroethylene; HCB, hexachlorobenzene; DDT, dichlorodiphenyltrichloroethane; PFOA, perfluorooctanoic acid; PFOS, perfluorooctanesulfonic acid; PFHxS, perfluorohexane sulfonate; PFHpS, perfluoroheptane sulfonate; PFNA, perfluorononanoic acid, PFDA, perfluorodecanoic acid; Al, aluminum; Ba, barium; As, arsenic; Cd, cadmium; Co, cobalt; Cu, copper; Mn, manganese; Mo, molybdenum; Sb, antimony; Ni, nickel; Se, selenium; Zn, zinc; TCDD, 2,3,7,8-tetrachlorodibenzo-p-dioxin; NO, nitric oxide; SO_2, sulfur dioxide; NAP, naphthalene; PHEN, phenanthrene; FLU, fluorene; PYR, pyrene.
